# A Ham1p-Dependent Mechanism and Modulation of the Pyrimidine Biosynthetic Pathway Can Both Confer Resistance to 5-Fluorouracil in Yeast

**DOI:** 10.1371/journal.pone.0052094

**Published:** 2013-10-04

**Authors:** Mattias Carlsson, Marie Gustavsson, Guo-Zhen Hu, Eva Murén, Hans Ronne

**Affiliations:** 1 Department of Microbiology, Swedish University of Agricultural Sciences, Uppsala, Sweden; 2 Department of Medical Biochemistry and Microbiology, Uppsala University, Uppsala, Sweden; Institute of Enzymology of the Hungarian Academy of Science, Hungary

## Abstract

5-Fluorouracil (5-FU) is an anticancer drug and pyrimidine analogue. A problem in 5-FU therapy is acquired resistance to the drug. To find out more about the mechanisms of resistance, we screened a plasmid library in yeast for genes that confer 5-FU resistance when overexpressed. We cloned five genes: *CPA1*, *CPA2*, *HMS1*, *HAM1* and *YJL055W*. *CPA1* and *CPA2* encode a carbamoyl phosphate synthase involved in arginine biosynthesis and *HMS1* a helix-loop-helix transcription factor. Our results suggest that *CPA1*, *CPA2*, and *HMS1* confer 5-FU resistance by stimulating pyrimidine biosynthesis. Thus, they are unable to confer 5-FU resistance in a *ura2* mutant, and inhibit the uptake and incorporation into RNA of both uracil and 5-FU. In contrast, *HAM1* and *YJL055W* confer 5-FU resistance in a *ura2* mutant, and selectively inhibit incorporation into RNA of 5-FU but not uracil. *HAM1* is the strongest resistance gene, but it partially depends on *YJL055W* for its function. This suggests that *HAM1* and *YJL055W* function together in mediating resistance to 5-FU. Ham1p encodes an inosine triphosphate pyrophosphatase that has been implicated in resistance to purine analogues. Our results suggest that Ham1p could have a broader specificity that includes 5-FUTP and other pyrimidine analogoue triphosphates.

## Introduction

5-Fluorouracil (5-FU) is one of the oldest anticancer agents, but is still widely used. It was developed in the 1950:ies and is used to treat of a wide variety of cancers such as colorectal cancers, breast cancers and cancers in the aerodigestive tracts [1]. To exert its cytotoxic action, 5-FU must be taken up by the cell and converted into FdUMP, FUTP or FdUTP [1]. FUTP is incorporated into RNA whiereas FdUTP causes genotoxic stress by misincorporation into DNA. FdUMP binds to thymidylate synthase (TS) which then forms of a stable inactive complex together with its coenzyme 5,10-methylene tetrahydrofolate. This inhibits *de novo* synthesis of dTMP and causes misincorporation of uracil into DNA, a process called thymineless death. Work in several organisms has further shown that the lethal effect of uracil misincorporation can be suppressed by mutations in uracil-DNA *N*-glycosylase [2]–[5]. It is thus not the presence of misincorporated uracil as such that kills the cell, but rather the many nicks that are created when the DNA repair machinery tries to remove large amounts of misincorporated uracil. The primary cause of the anti-proliferative action of 5-FU was long thought to be thymineless death due to inactivation of TS. However, recent work in the yeast *Saccharomyces cerevisiae* has provided evidence that effects on RNA metabolism contributes significantly to the cytotoxic effects of 5-FU [6]–[9].

A major problem in 5-FU therapy is the ability of clonally selected tumour cells to acquire resistance to the drug. Characterization of the mechanisms behind acquired drug resistance is therefore important in order to improve both diagnosis and treatment. One known cause of increased 5-FU resistance in cancer cells is overexpression of TS due to gene amplification [10]. Other resistance mechanisms are known to exist, but are still poorly understood. We reasoned that any mechanisms involving 5-FU uptake, 5-FU metabolism or general drug detoxification systems are likely to be conserved in other eukaryotes. We therefore carried out a plasmid library screen in yeast for genes that cause increased resistance to 5-FU when overexpressed.

Five resistance genes were cloned: *CPA1*, *CPA2*, *HMS1*, *HAM1* and *YJL055W*. *CPA1* and *CPA2* encode subunits of the carbamoyl phosphate synthetase CPSase A [11], and *HMS1* encodes a helix-loop-helix transcription factor [12]. We found that *CPA1*, *CPA2* and *HMS1* are unable to confer 5-FU resistance in a *ura2* mutant which lacks pyrimidine biosynthesis, and inhibit the uptake and incorporation into RNA of both uracil and 5-FU. This suggests that they confer 5-FU resistance by stimulating pyrimidine biosynthesis. In contrast, *HAM1* and *YJL055W* confer 5-FU resistance also in a *ura2* mutant, and selectively inhibit the incorporation into RNA of 5-FU, but not uracil. This suggests that the latter two genes funcion in a resistance mechanism that does not depend on an increased pyrimidine biosynthesis. Consistent with this, *HAM1* encodes a nucleoside triphosphate pyrophosphatase that hydrolyzes non-canonical purine nucleotides [13]. Our finding suggests that the Ham1p enzyme may have a broader specificity, mediating resistance to 5-FU and other pyrimidine analogoues.

## Results

### Cloning of yeast genes that confer resistance to 5-FU when overexpressed

**Figure 1:**
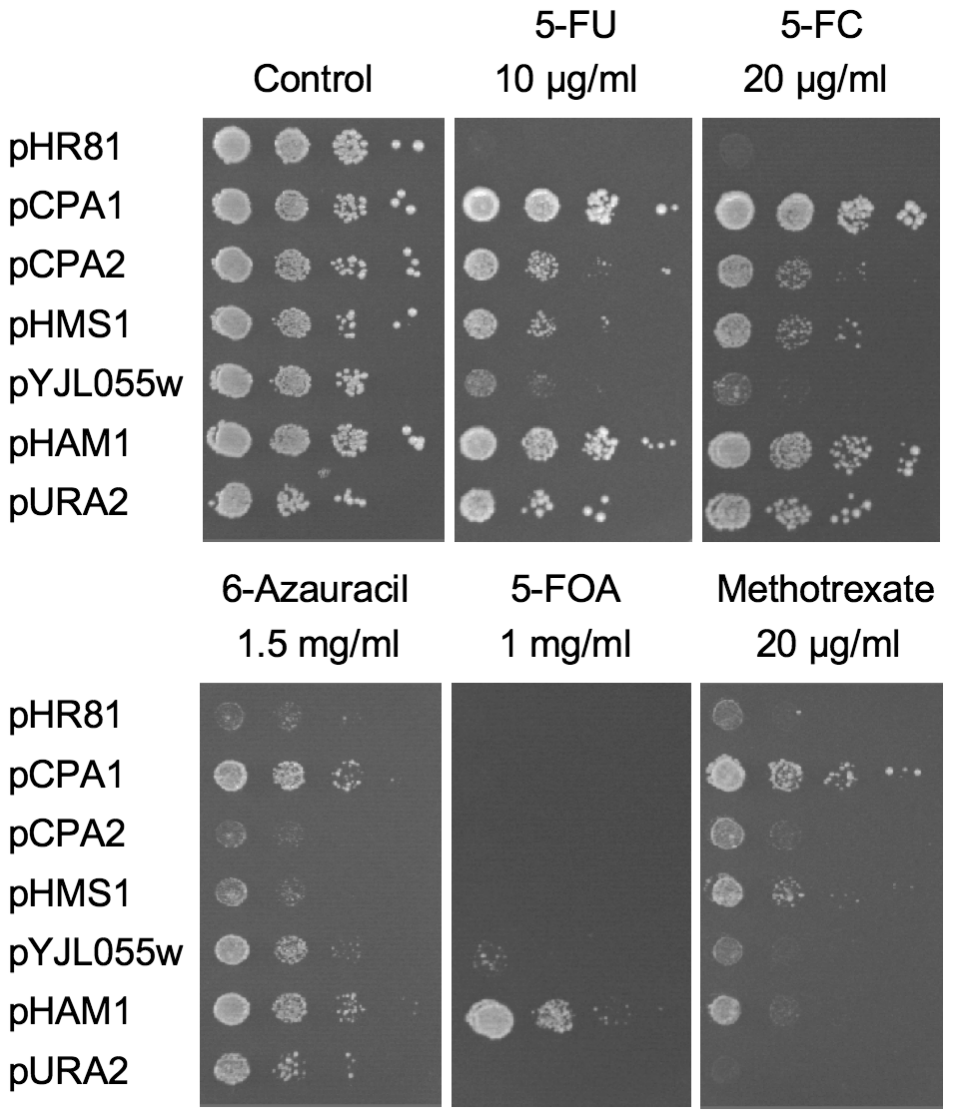
Drug resistance of yeast colonies containing different plasmids. The drugs tested were 5-FU, 5-FC, 6-azauracil, 5-FOA, and methotrexate in combination with sulfanilamide. Yeast cells were serially diluted and spotted onto uracil-less synthetic media with or without drugs at indicated concentrations. Growth was scored after incubation at 30°C for four days.

A yeast genomic DNA library made in the 2 µm *URA3 LEU2-d* vector pHR81 [14] was transformed into the wild type yeast strain BY4742, and screened for transformants that could grow in the presence of 40 µg/ml of 5-FU. After rescue of the plasmids into *E. coli* and retesting in yeast, we found nine positive clones, and mapping of the inserts identified five genes that confer resistance to 5-FU: *CPA1*, *CPA2*, *HMS1*, *HAM1* and *YJL055W* (Fig. S1). The strongest resistance genes are *CPA1* and *HAM1*, followed by *CPA2* and *HMS1*, whereas *YJL055W* has a much weaker effect (Fig. 1).

Cpa1p and Cpa2p are the two subunits of the carbamoyl phosphate synthetase CPSase A, which functions in arginine biosynthesis [11]. Cpa1 uses glutamine to produce ammonia, which is then used by Cpa2p to make carbamoyl phosphate, the starting compound for both arginine and pyrimidine biosynthesis. A distinct pyrimidine biosynthesis-specific carbamoyl phosphate synthase is encoded by the *URA2* gene [15]. We therefore also tested if *URA2* causes 5-FU resistance when overexpressed. As shown in Fig. 1, this is indeed the case. We conclude that overproduction of either carbamoyl phosphate synthase causes 5-FU resistance. This is consistent with the carbamoyl phosphate pool in yeast being freely exchangeable between the two biosynthetic pathways [15].

*HMS1* was first cloned as a high copy number suppressor of the filamentation growth defect of a *mep1 mep2* double mutant [12]. It encodes a myc-like helix–loop–helix protein [16]. Its physical interaction with Pcl1p, a G_1_ cyclin, suggests that Hms1p may regulate the mitotic exit machinery [17]. Several possible target genes for Hms1p have been identified by phenotypic activation [18]. The *YJL055W* gene is known to confer resistance to 5-FOA when overexpressed, and further testing showed that it also confers resistance to 5-FU [19]. Conversely, a *yjl055w* mutant is sensitive to purine analogs [20]. It has therefore been proposed that *YJL055W* may function in detoxification of base analogs [19].

The fifth gene, *HAM1* encodes a nucleotide phosphatase that targets non-canonical purine nucleotides such as ITP, dITP, XTP and dXTP [13], [21]. A *HAM1* orthologue is present in all eukaryotes, archaeotes and bacteria that have been examined, indicating that it provides a ancient and highly conserved function. The yeast gene was originally identified as a mutant sensitive to the mutagen 6-N-hydroxylaminopurine (HAP), hence its name [22]. Significantly, a missense mutation in the human orthologue, IPTA, has been linked to increased sensitivity to mercaptopurine, a purine analogue used in treatment of acute lymphoblastic leukemia [23]–[24]. Recent work has further shown that IPTA is important for maintaining genome stability and the prevention of apoptosis in human cells [25].

### Effects of the cloned genes on the resistance to other drugs

To examine the specificity of the drug resistance conferred by the cloned genes, we tested the overexpression strains for resistance to three other pyrimidine analogues: 5-fluoro orotic acid (5-FOA), 5-fluorocytosine (5-FC) and 6-azauracil. We also tested methotrexate, which inhibits dihdrofolate reductase and thus indirectly targets thymidine synthase, a target of 5-FU. As shown in Fig. 1, we found that resistance to 5-FU and 5-FC had similar patterns, with *CPA1* and *HAM1* being the strongest resistance genes, and *YJL055W* by far the weakest. In contrast, 5-FOA resistance was only conferred by *HAM1*, and weakly also by *YJL055W* (Fig. 1). For 6-azauracil, we found that *CPA1* and *HAM1* were the two strongest resistance genes, as for 5-FU and 5-FC. However, *YJL055W* had a stronger effect and *CPA2* and *HMS1* a weaker effect on resistance to 6-azauracil than on resistance to 5-FU and 5-FC. Resistance to methotrexate exhibited a different pattern (Fig. 1). In this case, *CPA1* was the strongest resistance gene, with *HMS1* and *HAM1* conferring a weaker effect, and the other genes, in particular *URA2*, being largely ineffective.

### Effect of knockout mutations on 5-FU sensitivity

**Figure 2:**
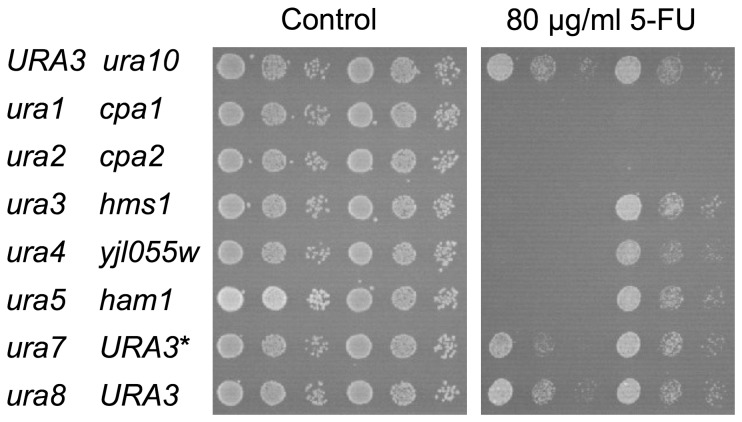
Sensitivity of pyrimidine biosynthesis mutants to 5-FU. All mutants tested were *kanMX* gene disruptions in the BY4742 genetic background. Since the BY4742 background is *ura3*, all strains except the *ura3* strain were first transformed with the *HIS3 URA3* plasmid pHGZ252 in order to restore *URA3* function. The *ura3* strain is BY4742 transformed with the *HIS3* plasmid pRS413. *URA3* stands for BY4742 with a restored wild type *URA3* locus transformed with with pHGZ252, included as a control. URA3* is BY4742 transformed with pHGZ252, included as an additional control. Cells grown in histidine-less media were diluted 10-fold in water and spotted onto synthetic complete plates in the presence or in the absence of 80 µg/ml of 5-FU. Growth was scored after incubation at 30°C for three days.

To test if a deletion of our cloned genes would have the opposite effect of overexpression, we assayed the corresponding knockout strains for sensitivity to 5-FU. Several knockouts affecting different steps in the pyrimidine biosynthesis were also tested. Since most of the strains carry a *ura3* mutation in addition to the mutations we wanted to test, and since *ura3* confers sensitivity to 5-FU (Fig. S2), all strains were first made *URA3^+^* by transformation with plasmid pHGZ252, that contains the *URA3* and *HIS3* genes, with the latter being used to select transformants. As shown in Fig. 2, we found that a deletion of either *CPA1*, *CPA2* or *URA2* confers a significantly increased sensitivity to 5-FU. A likely interpretation is that a reduced synthesis of carbamoyl-P in these mutants reduces pyrimidine biosynthesis, which makes the cells more sensitive to 5-FU. In contrast, a deletion of *HMS1*, *YJL055W* or *HAM1* has no apparent effect on 5-FU sensitivity (Fig. 2).

Deletions of *URA1*, *URA4* or *URA5* also cause increased 5-FU sensitivity, while deletions of *URA7*, *URA8* or *URA10* have no such effect. A likely reason for the lack of effect in the latter case is that *URA10* encodes a minor isozyme, while *URA7* and *URA8* encodes CTP synthase, the loss of which should not affect UTP synthesis. In contrast, *URA1* and *URA4* are single copy genes, and *URA5* encodes a major isozyme that accounts for most of the activity in the same biosynthetic step as Ura10p.

### Interactions between the resistance genes

In a search for functional relationships between the cloned genes, we tested to what extent the ability of each gene to confer resistance to 5-FU depends on any of the other genes. Each plasmid was thus transformed into knockout mutants for the other five genes and tested for effects on 5-FU sensitivity. As shown in Fig. S3, we found several cross-dependencies. Thus, *CPA1* depends on *CPA2*, *CPA2* depends partially on *CPA1*, and *HMS1* and *YJL055W* depend on both *CPA1* and *CPA2*. In addition, *HAM1* depends partially on *CPA1*, *CPA2* and also on *YJL055W* (Fig. S3). Since the *ura2* mutant does not grow on uracil-less media, experiment with *ura2* cells were carried out on synthetic complete medium, using a higher concentration of 5-FU since the drug is less effective in the presence of uracil. We further tested if the 5-FU resistance conferred by *URA2* depends on any of the other genes. As shown in Fig. S3 and S4, none of the other genes are required for *URA2* to confer 5-FU resistance.

### Dependency of resistance genes on pyrimidine biosynthesis

**Figure 3:**
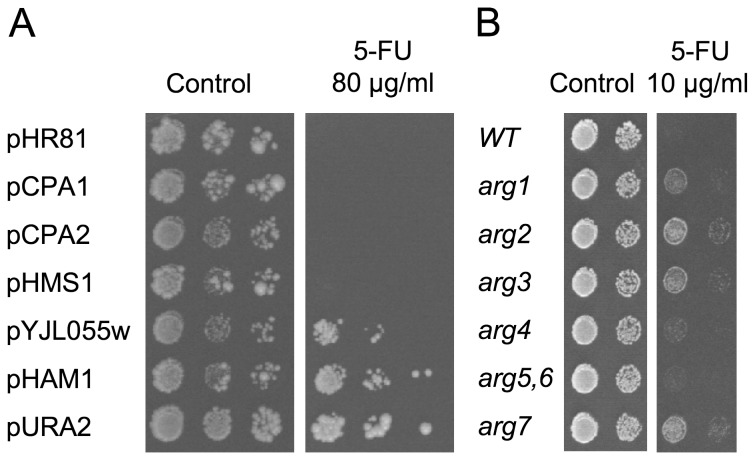
Genetic interactions affecting 5-FU sensitivity. (A) Dependency of the 5-FU resistance conferred by different plasmids on *URA2* function. A *ura2* strain containing different plasmids was grown to late exponential phase in synthetic uracil-less medium supplemented with 1 g/l of orotic acid, serially diluted, and spotted onto plates with or without 80 µg/ml of 5-FU. (B) Effect of arginine biosynthesis mutants on 5-FU sensitivity. Since the deletion mutants were made in BY4742, which is *ura3*, all strains were transformed with the *URA3* plasmid pHR81 in order to restore a functional pyrimidine biosynthesis. Transformants were grown in liquid medium to late exponential phase, serially diluted, and spotted onto synthetic uracil-less plates with or without 10 µg/ml of 5-FU. Growth was scored after incubation at 30°C for four days.

A key question is to what extent different resistance genes depend on the pyrimidine biosynthesis. To address this question, we tested the plasmids in a *ura2* knockout mutant. Ura2p is a bifunctional enzyme that catalyzes two consecutive steps in the pyrimidine biosynthesis: carbamoyl-P synthesis and conversion of carbamoyl-P to carbamoyl-aspartate. A *ura2* mutant is therefore unable to synthesize pyrimidines even if carbamoyl-P is provided by the Cpa1p/Cpa2p enzyme. As shown in Fig. 3A, we found that *CPA1*, *CPA2* and *HMS1* are strictly dependent on *URA2* for their ability to confer 5-FU resistance. In contrast, *YJL055W* and *HAM1* are at least partially effective also in the *ura2* mutant. From this we conclude that *CPA1*, *CPA2* and *HMS1* require pyrimidine biosynthesis to confer 5-FU resistance. This is consistent with the notion that these three genes confer 5-FU resistance by stimulating the *de novo* pyrimidine biosynthesis and thus diluting the drug. Conversely, the fact that *YJL055W* and *HAM1* can confer 5-FU resistance even in the absence of pyrmidine biosynthesis suggests that these two genes cause resistance in some other way.

The main target for *in vivo* regulation of CPSase A is *CPA1*. We therefore tested if any of the other resistance genes would induce *CPA1* expression, using reverse transcriptase PCR. As expected, we found that the *CPA1* mRNA is overproduced in the presence of the *CPA1* plasmid (Fig. S5). However, we saw no evidence that any other gene induces *CPA1* expression. On the contrary, *CPA1* expression is reduced in the presence of the *HMS1* plasmid. This could, however, be due to the fact that the *HMS1* plasmid inhibts cell growth, thus causing a secondary downregulation of pyrimidine biosynthesis.

### Arginine repression of 5-FU resistance

**Figure 4:**
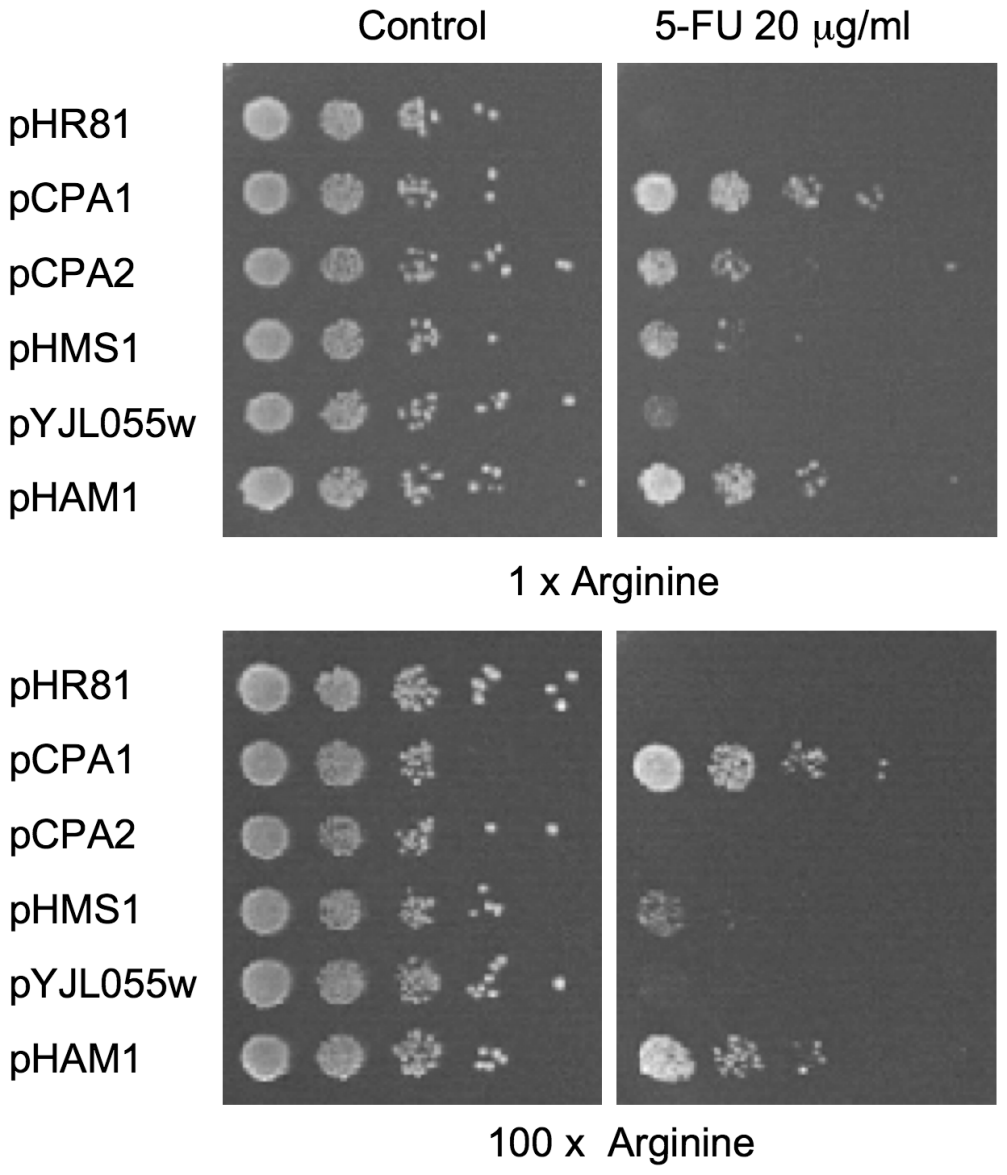
Effect of excess arginine on 5-FU resistance conferred by different plasmids. Ten-fold serial dilutions of yeast strains containing different plasmids were plated onto proline-based synthetic complete media containing 20 µg/ml 5-FU and either the normal amount of arginine (0.1 mM), or a 100-fold excess of arginine (10 mM).

The leader of the *CPA1* mRNA contains an upstream ORF encoding a 25-amino acid peptide. In the presence of arginine, this peptide represses translation of the *CPA1* mRNA [26]. In addition, transcription of the *CPA1* mRNA is also inhibited in the presence of arginine. Since several of the genes were dependent on *CPA1* for their abilities to confer 5-FU resistance, we proceeded to test all five cloned overexpression plasmids in the presence of a 100-fold excess of arginine. As shown in Fig. 4, we found that *CPA1* itself can confer 5-FU resistance also in the presence of high concentrations of arginine. This is not surprising, since overexpression of *CPA1* is likely to override its own transcriptional and translational repression by arginine. However, we further found that the 5-FU resistance conferred by *CPA2* is completely inhibited by a 100-fold excess of arginine, and that the resistance conferred by *HMS1* is partly inhibited (Fig. 4). This is consistent with these two genes primarily conferring 5-FU resistance by stimulating the pyrimidine biosynthesis, which explains why their effects are sensitive to inhibition of *CPA1* by arginine. In contrast, the ability of *HAM1* to confer 5-FU resistance was not affected by arginine repression (Fig. 4), which supports the notion that it acts through a different mechanim. For *YJL055W*, finally, the effect of arginine was difficult to assess due to its much weaker 5-FU resistance.

### Interactions with mutations in the arginine biosynthetic pathway

The cross-talk between the pyrimidine and arginine biosynthetic pathways prompted us to test mutations in the arginine biosynthetic pathway for effects on 5-FU sensitivity. The rationale for this was that we wanted to see if a reduced arginine biosynthesis might channel carbamoyl-P into pyrimidine biosynthesis, and thus confer 5-FU resistance. As shown in Fig. 3B, we found that this is indeed the case. Thus, *arg2*, *arg3* and *arg7* mutants are clearly more resistant to 5-FU than the wild type strain, and a weaker effect is seen in the *arg1* mutant. In contrast, the *arg4* and *arg5,6* mutants did not show any clear effect on 5-FU resistance.

Arg3p, Arg1p and Arg4p catalyse consecutive steps in the pathway from condensation of ornithine and carbamoyl-P (Arg3p) to arginine formation (Arg4p). It therefore makes sense that a mutation in the first step, *arg3*, which blocks all carbamoyl-P consumption, has the strongest effect on 5-FU resistance, while mutations further downstream have a smaller effect (*arg1*) or no effect at all (*arg4*). The other three mutants that were tested, *arg2*, *arg5,6* and *arg7*, are all involved in the formation of ornithine from glutamate. If ornithine formation is blocked, this will prevent the use of carbamoyl-P in arginine biosynthesis and instead shunt it into pyrimidine biosynthesis. The fact that *arg2* and *arg7* are resistant to 5-FU is consistent with this notion. However, the fact that the *arg5,6* mutant is not resistant suggests that some mechanism exists that can bypass the need for Arg5,6p in ornithine formation, or alternatively that the *arg5,6* mutant also interferes with channelling of excess carbamoyl-P into pyrimidine biosynthesis.

### Effects on the uptake and incorporation into RNA of uracil and 5-FU

If the 5-FU resistance conferred by a gene is due to an increased pyrimidine biosynthesis, we should expect to see effects on the uptake and incorporation into RNA of externally added uracil and 5-FU. We therefore proceeded to study this. Yeast cells grown to mid exponential phase were labelled for 60 min with either [^14^C]uracil or [^14^C]5-FU, and the uptake of either compound was then determined in a scintillation counter. RNA was extracted and separated on polyacrylamide gels in urea, after which autoradiography was used to quantify the label incorporated into tRNA. tRNA was chosen because it forms a distinct spot on the gels that can be easily quantified.

**Figure 5:**
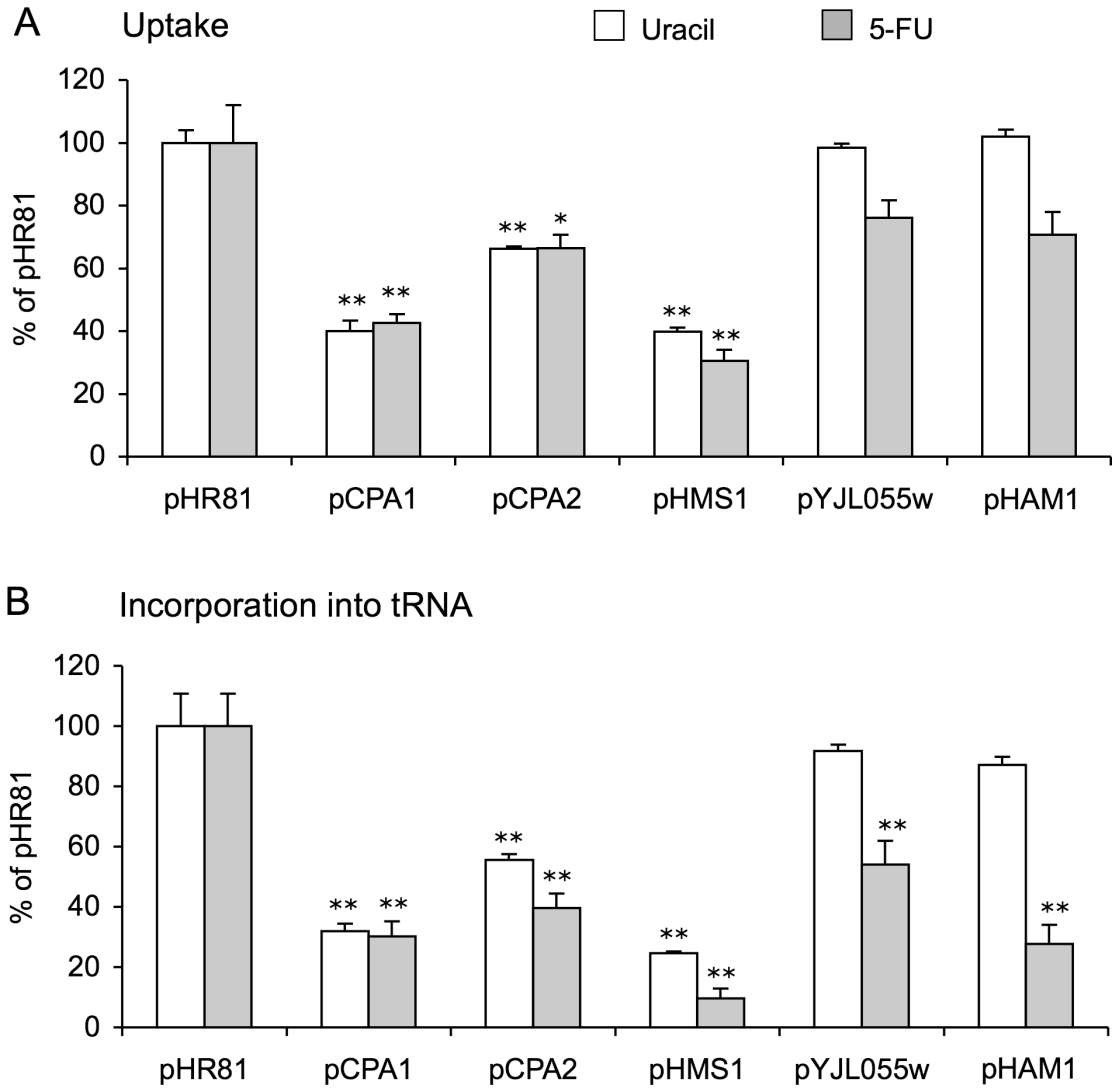
Effect of plasmids on the metabolism of 5-FU and uracil. (A) Uptake of ^14^C-labelled uracil or 5-FU. (B) Incorporation of ^14^C-labelled uracil or 5-FU into tRNA. Yeast cells containing different plasmids, as indicated in the figure, were incubated with ^14^C-labelled uracil or 5-FU. Uptake of the ^14^C-labelled compounds and their incorporation into tRNA was quantified as described in Materials and Methods. The error bars show standard errors of the mean in experiments performed in quadruplicate. Bars with two asterisks differ significantly from the pHR81 value at p<0.01 and bars with one asterisk at p<0.05.

As shown in Fig. 5, we found that the resistance genes fall into two distinct groups. Thus, *CPA1*, *CPA2* and *HMS1* reduce the uptake and incorporation into tRNA of both uracil and 5-FU. These effects were all significant at p<0.01 except for the effect of *CPA2* on the uptake of 5-FU, which was significant at p<0.05. These findings are consistent with the notion that these three genes cause 5-FU resistance by increasing the *do novo* synthesis of pyrimidines. In contrast, *HAM11* and *YJL055W* have no effect on either the uptake or incorporation into tRNA of uracil. However, both genes significantly (p<0.01) reduce incorporation into tRNA of 5-FU (Fig. 5B). Both genes also had a small effect on the uptake of 5-FU (Fig. 5A) but this effect was not significant. We conclude that *HAM1* and *YJL055W* primarily act to reduce the amount of 5-FU that is incorporated into RNA, and that this effect is specific for 5-FU and does not affect uracil.

## Discussion

**Figure 6:**
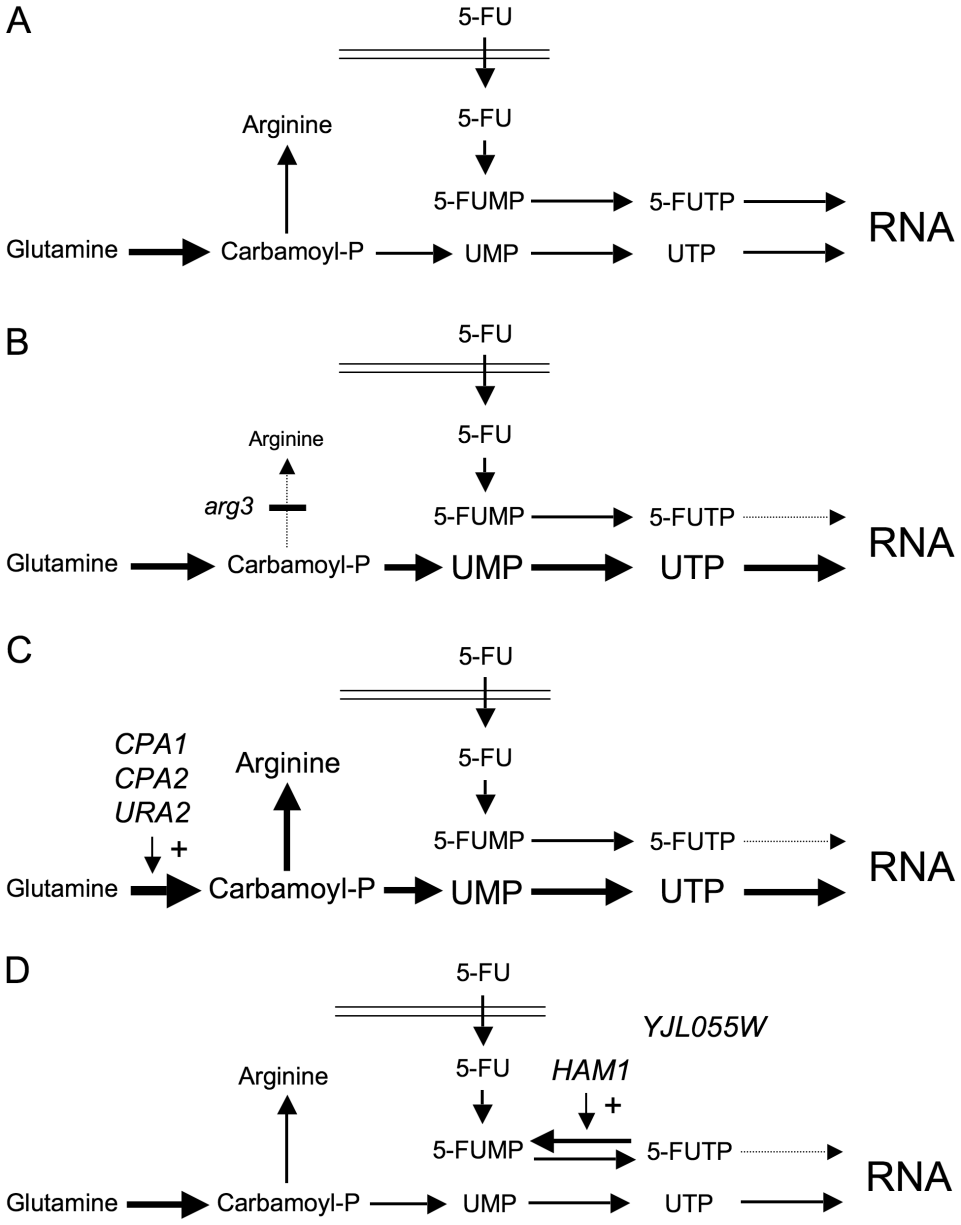
Schematic overview of the pyrimidine biosynthetic pathway and its effect on the sensitivity of yeast cells to 5-FU under different conditions. (A) Wild type cells. 5-FU is taken up and converted to 5-FUTP, which competes with freshly synthesized UTP for incorporation into RNA. (B) In an arginine biosynthesis mutant, more carbamoyl-P is shunted into the pyrimidine biosynthesis, and the resulting increased in UTP reduces the amount of 5-FUTP that is incorporated into RNA. (C) In cells overexpressing *CPA1*, *CPA2* or *URA2*, more carbamoyl-P is made, which also results in an increase in UTP that reduces the incorporation of 5-FUTP into RNA. (D) In cells overexpressing *HAM1*, the amount of 5-FU that is incorporated into RNA is reduced by dephosphorylation of 5-FUMP back to 5-FUTP. Genetic interactions suggest that *YJL055W* also may affect this process.

We have identified six genes whose overexpression confer resistance to the anticancer drug 5-FU in yeast. Three genes, *CPA1*, *CPA2*, and *URA2*, encode subunits of the two carbamoyl phosphate synthetases [11]. A likely explanation why they confer resistance to 5-FU is that increased production of carbamoyl phosphate stimulates pyrimidine biosynthesis. This will dilute the 5-FU and decrease its cytotoxic effect (Fig. 6C). This interpretation is supported by our finding that mutations which block pyrimidine biosynthesis confer sensitivity to 5-FU (Fig. 2), and by the observation that a constitutively active *URA2* mutant is 5-FU resistant [27]. Conversely, we found that mutations in the arginine biosynthesis confer resistance to 5-FU (Fig. 3B). A likely explanation is that these mutations stimulate pyrimidine biosynthesis by shunting carbamoyl phosphate into the latter pathway (Fig. 6B). That modulation of the pyrimidine biosynthesis can affect the sensitivity to 5-FU is also supported by observations in other organisms. Thus, a 5-FU resistant *Salmonella* mutant had an increased CPSase activity [28], and phaseolotoxin, which inhibits ornithine transcarbamylase, confers resistance to 5-FU in plant cells [29]. Furthermore, uridine protects against 5-FU cytotoxicity in mammalian cells [30], and two 5-FU resistant mutants in *Aspergillus* were found to affect the arginine biosynthesis [31].

Our results raise the possibility that an increase in CPSase activity due to amplification or upregulation of CPSase genes could play a role in acquisition of 5-FU resistance by cancer cells. If so, CPSase could be used as a target for anticancer therapy, and as a marker to detect 5-FU resistant tumours. Similar to yeast, animals have two carbamoyl phosphate synthases, CPS I and CPS II, which function in arginine and pyrimidine biosynthesis, respectively. However, unlike yeast where both enzymes are cytosolic, CPS I resides in the mitochondria [32]–[34]. Furthermore, CPS I is primarily expressed in the liver, where it functions in the urea cycle. CPS II activity is known to be increased in several human cancers, particularly in rapidly growing tumours [35]. However, this could be a secondary effect of an increased pyrimidine biosynthesis in replicating cells. A more relevant question is therefore if CPS II (or CPS I) is specifically overproduced in 5-FU resistant cell lines. Some data suggest that this may be the case. Thus, RNA expression profiling of normal and 5-FU resistant colon cancer cell lines showed that CPS II was upregulated 1.8-fold in the latter [36].

Our results suggest that *HMS1* also confers 5-FU resistance by affecting the pyrimidine biosynthesis. We base this conclusion on two observations. First, *HMS1* resembles *CPA1* and *CPA2* in being dependent on *URA2* (Fig. 3A). Second, it resembles *CPA1* and *CPA2* in that it reduces the uptake and incorporation of uracil and 5-FU to the same degree (Fig. 5). The fact that Hms1p is a transcription factor suggests that it could act by regulating some other gene(s) with an effect on pyrimidine biosynthesis. We checked one obvious candidate, *CPA1*, but could see no effect of the *hms1* knockout, nor did overexpression of *HMS1* increase *CPA1* expression (Fig. S4). Genes that respond to overexpression of different transcription factors, including Hms1p, have been identified [18]. The data on Hms1p were hard to interpret, but none of the genes known to be involved in pyrimidine biosynthesis was strongly induced by its overexpression. However, studies using different nitrogen sources may be needed to reveal any such regulation. Interestingly, *HMS1* was originally cloned as a suppressor of mutations in the nitrogen-repressed ammonium permase genes *MEP1* and *MEP2* [12], which suggests a possible role in the control of nitrogen uptake. It is conceivable that an increased nitrogen uptake could stimulate pyrimidine biosynthesis if the latter is limited by nitrogen availability.

Unlike the other four genes, *HAM1* and *YJL055W* do not seem to stimulate pyrimidine biosynthesis. We base this conclusion on three observations. First, both genes confer 5-FU resistance also in the *ura2* strain (Fig. 3A). Second, the 5-FU resistance conferred by *HAM1* is not sensitive to arginine repression (Fig. 4). Third, *YJL055W* and *HAM1* specifically reduce the incorporation into RNA of 5-FU, but have no effects on either the uptake or incorporation into RNA of uracil (Fig. 5). The latter finding suggests that *YJL055W* and *HAM1* affect a process that is specific for 5-FU (Fig. 6D). As discussed below, a likely mechanism is dephosphorylation of 5-FUTP to 5-FUMP. We further note that a role for *YJL055W* and *HAM1* in drug detoxification is consistent with the resistance profiles in Fig. 1. Thus, all six plasmids conferred resistance to 5-FU and 5-FC, and the profiles were very similar for these two drugs. In contrast, *HAM1*, and to some extent *YJL055W*, is more efficient against 5-FOA than the other genes. *YJL055W* was also more efficient against 6-azauracil than against 5-FU and 5-FC. It thus seems that *HAM1* and *YJL055W* have broader specificities than the other resistance genes. *HAM1* is also partly dependent on *YJL055W*, unlike the other resistance genes (Fig. S3). This supports the notion that a distinct mechanism for 5-FU resistance exists which involves Ham1p and Yjl055wp. However, our finding that a deletion of neither gene causes a significantly increased sensitivity to 5-FU (Fig. 2) suggests that this mechanism is redundant with some other detoxification mechanism under normal conditions. The nature of that mechanism remains to be determined, but it is unlikely to involve increased uracil synthesis, as the *ham1* deletion did not confer an increased sensitivity to 5-FU in a *ura3* strain supplemented with uracil (data not shown).

*HAM1* is the most potent resistance gene, and unlike *YJL055W* it is also conserved in all organisms studied. Work in several organisms have shown that the encoded protein is a nucleoside triphosphate pyrophosphohydrolase that is active against several noncanonical purine triphosphates [13], [21]–[24]. Moreover, a study in yeast showed that overexpression of *HAM1* detoxifies 5-bromodeoxyuridine, indicating that the enzyme may be active also against pyrimidines [37]. Our results extend this finding to 5-FU, 5-FC, 6-azauracil and 5-FOA (Fig. 1), and suggests that a wide range of noncanonical pyrimidine triphosphates may be targeted by Ham1p and its human homolog, *ITPA*. This raises the possibility that amplification and/or overexpression of *ITPA* may contribute to acquired resistance to 5-FU and other pyrimidine analogues in tumour cells.

## Materials and Methods

### Yeast strains and plasmids

Yeast deletion strains in the BY4742 background were obtained from the Euroscarf collection (http://www.uni-frankfurt.de/fb15/mikro/euroscarf). The open reading frame in each deletion strain has been replaced by the *KanMX* selection cassette. The *URA3* strain H1634 was created by integration of the wildtype *URA3* gene into the *ura3Δ* locus of BY4742. Plasmid pURA2 was made by cloning a 7972 bp *Sal*I-*Kpn*I fragment of pFL39URA2 (kindly provided by Francois Lacroute) containing the *URA2* gene between the *Sal*I and *Kpn*I sites of the *URA3* 2 µm vector pFL44 [38]. The *URA3 HIS3* plasmid pHGZ252 was made by cloning an 1150 bp fragment carrying the *HIS3* gene into the unique *Sma*I site of pJK101 [39].

### Growth media and chemicals

Rich media (YPD) and synthetic complete (SC) or dropout media were prepared as previously described [40]. Arginine repression media used equimolar amounts of proline instead of ammonium sulphate, since Cpa2p can use ammonium ions instead of glutamine, thus making it independent of Cpa1p [11]. Use of a non-ammonium nitrogen source is therefore needed to detect repression of *CPA1*. 5-FU dissolved in 50 mg/ml glucose was obtained from Apoteksbolaget (Uppsala, Sweden). 6-Azauracil was obtained from MP biochemicals (Illkirch, France). 5-Fluoroorotic acid (5-FOA), monensin, orotic acid, sulfanilamide and methotrexate were obtained from Sigma-Aldrich (Stockholm, Sweden). 5-Fluorocytosine was obtained from Apollo Scientific Ltd (Bredbury, UK).

### Yeast transformation and growth

Yeast cells transformed with a yeast genomic library made in the 2 µm *URA3 LEU2-d* vector pHR81 [14] were selected on synthetic uracil-less media, as previously described [40]. For the *ura2* strain, transformants were selected on acidic (pH 2.7) synthetic uracil-less media containing 1 g/l of orotic acid. Orotic acid is an intermediate in the pyrimidine biosynthesis between the Ura2p and Ura3p enzymes, and the ability to use orotic acid can therefore be used to select for the *URA3* marker in a *ura2* strain. To assay drug sensitivity, transformants were grown overnight at 30°C in synthetic uracil-less media, supplemented with 1 g/l of orotic acid in the case of the *ura2* strain. These overnight precultures were diluted into fresh media to a final OD_600_ of 0.1 and grown to late exponential phase. Cells were harvested and serial 10-fold dilutions in water were prepared. A 5 µl aliquot of each dilution was spotted onto control plates and drug plates. Growth was monitored after two days at 30°C.

### Plasmid library screen

The wild type strain BY4742 was transformed with a yeast genomic DNA library made in the *URA3* vector pHR81 [14]. Transformants were selected on synthetic media lacking uracil. After three days growth at 30°C approximately 50,000 transformants were replicated onto synthetic media lacking uracil but containing 40 µg/ml of 5-FU. The plates were monitored for 5-FU resistant clones, which were picked after 5 and 7 days. Plasmids were rescued from these clones, retransformed into BY4742 and retested for 5-FU resistance. The genes responsible for 5-FU resistance were mapped by deletions and/or PCR subcloning, followed by retesting of the resulting plasmids in yeast.

### Quantification of uptake and incorporation into RNA of uracil and 5-FU

BY4742 cells transformed with different plasmids were grown in 10 ml synthetic medium lacking uracil and leucine to an OD_600_ of 0.5. Leucine selection was used to force a high copy number, taking advantage of the defective *LEU2-d* marker on the pHR81 vector [14]. The cells were then diluted to an OD_600_ of 0.2 and incubated with 0.25 mCi ml^−1^ of either [^14^C]uracil or [^14^C]5-FU (50–60 mCi/mmol; Larodan) for 60 minutes, after which total RNA was extracted [41]. Aliquots of 20 µl of washed cells suspended in 400 µl TES buffer were saved for measurement of [^14^C]uracil and [^14^C]5-FU uptake. The aliquots were diluted to 750 µl in water and the OD_600_ was measured to provide an estimate of the number of cells. The cell suspensions were added to 2 ml of Optiphase hisafe 3 (Perkin Elmer, USA) after which the radioactivity in each sample was measured in a Beckman Coulter LS 6000IC scintillation counter. RNA concentrations in extracts were measured using a GeneQuant Pro system. Aliquots of 3.5 µg of [^14^C]5-FU-labeled RNA or 1 µg of [^14^C]uracil-labeled RNA were separated on 5% polyacrylamide gels containing 7 M urea (BioRad) in Tris-Borate-EDTA buffer by electrophoresis at 70 V for 1 h and 10 min. The RNA was visualized by ethidium bromide staining, and quantified using the Quantity One 4.5.0 software in a BioRad Gel Doc EQ System. Incorporated ^14^C-label was quantified by gel autoradiography using the Fujifilm BAS-2500 system. Specific ^14^C incorporation into tRNA was calculated as the ratio between the ^14^C autoradiographic signal and the ethidium bromide fluorescence signal. All values were normalized against the results obtained with the empty cloning vector pHR81.

### Reverse transcriptase-PCR quantification of mRNA

Total RNA was prepared from yeast as described by Ausubel et al. [41]. Samples of 2 µg of RNA were treated with RNase-free DNase I (Fermentas). Reverse transcription reactions were performed using a First Strand cDNA synthesis kit from Fermentas. The *CPA1* mRNA was amplified using the primer pair MC20 (CAA ATG TCC TCC GCT GCA AC) and MC21 (ATA GCT GTG TCT AAG GGA CC), and the *ACT1* mRNA using the primer pair MC24 (CGT TCC AAT TTA CGC TGG TT) and MC25 (CGG TGA TTT CCT TTT GCA TT). The PCR products were separated on agarose gels and visualized by ethidium bromide staining.

## Supporting Information

Figure S1 **Restriction maps of inserts of the plasmids isolated in the 5-FU resistance screen.** Open reading frames are shown as boxes. Below each insert, the shortest subclone that could still confer 5-FU resistance when overexpressed is shown.
(TIF)

Figure S2 **Effects of** ***URA3*** **copy number on 5-FU sensitivity.** Strains tested included the *ura3* yeast strain BY4742, a *URA3* transformant of BY4742 that carries the wild type gene at the *URA3* locus, and BY4742 containing either the low copy number centromeric *URA3* plasmid pFL39 or the high copy number 2 µm *URA3* plasmid pFL44. The strains were grown in liquid medium to late exponential phase, serially diluted, and spotted onto synthetic complete media with or without 5-FU. Growth was scored after incubation at 30°C for four days.
(TIF)

Figure S3 **Cross-dependencies between different genes for the ability to confer 5-FU resistance.** Each plasmid was transformed into yeast knockout strains where one of the other resistance genes had been deleted. Transformants were grown in liquid medium to late exponential phase, serially diluted, and spotted onto uracil-less plates with or without 5-FU at the indicated concentrations. In the bottom panes, synthetic complete media was used in order to permit growth of the control *ura2* strain. Note that ammonium sulphate was used as nitrogen source, hence the dependency of *CPA2* on *CPA1*, which provides ammonium ions to the *CPA2* encoded enzyme, is only weakly detectable.
(TIF)

Figure S4 **Test for dependencies of other genes on** ***HAM1*** **for the ability to confer 5-FU resistance.** Each plasmid was transformed into *ham1* knockout and wild type strains. Transformants were grown in liquid medium to late exponential phase, serially diluted, and spotted onto uracil-less plates with or without 5-FU at the indicated concentrations. pHAM1-1 is a PCR subclone of pHAM1 containing sequences from 414 bp upstream of the *HAM1* open reading frame to 301 bp downstream of the openreading frame.
(TIF)

Figure S5 **Analysis of** ***CPA1*** **expression by reverse transcriptase-PCR.** RNA was prepared from different knockout strains and the wild type BY4742 strain harbouring different plasmids. The RNA was used for reverse transcriptase-PCR of the *CPA1* mRNA as described in Materials and Methods. The PCR products were separated on an agarose gel and visualized by ethidium bromide staining. As a control, we included the *ACT1* mRNA encoding yeast actin. Lanes: 1, wild type; 2, *cpa1* strain; 3, *cpa2* strain; 4, *hms1* strain; 5, *yjl055w* strain; 6, pHR81 (empty vector); 7, pCPA1; 8, pCPA2; 9, pHMS1; 10, pYJL055W; 11, pHAM1.
(TIF)
