# Correction: A Ham1p-Dependent Mechanism and Modulation of the Pyrimidine Biosynthetic Pathway Can Both Confer Resistance to 5-Fluorouracil in Yeast

**DOI:** 10.1371/annotation/fe863e39-ab49-40e2-b262-0219428de65e

**Published:** 2013-11-26

**Authors:** Mattias Carlsson, Marie Gustavsson, Guo-Zhen Hu, Eva Murén, Hans Ronne

This article was republished on November 20, 2013, to replace an incorrectly published version. Please download this article again to view the correct version. 

