# Supplementary figures and images for: A Ham1p-Dependent Mechanism and Modulation of the Pyrimidine Biosynthetic Pathway Can Both Confer Resistance to 5-Fluorouracil in Yeast
Source: PLoS One. 2013 Oct 4;8(10):e52094. doi: 10.1371/journal.pone.0052094 (PMC3792807; doi:10.1371/journal.pone.0052094)

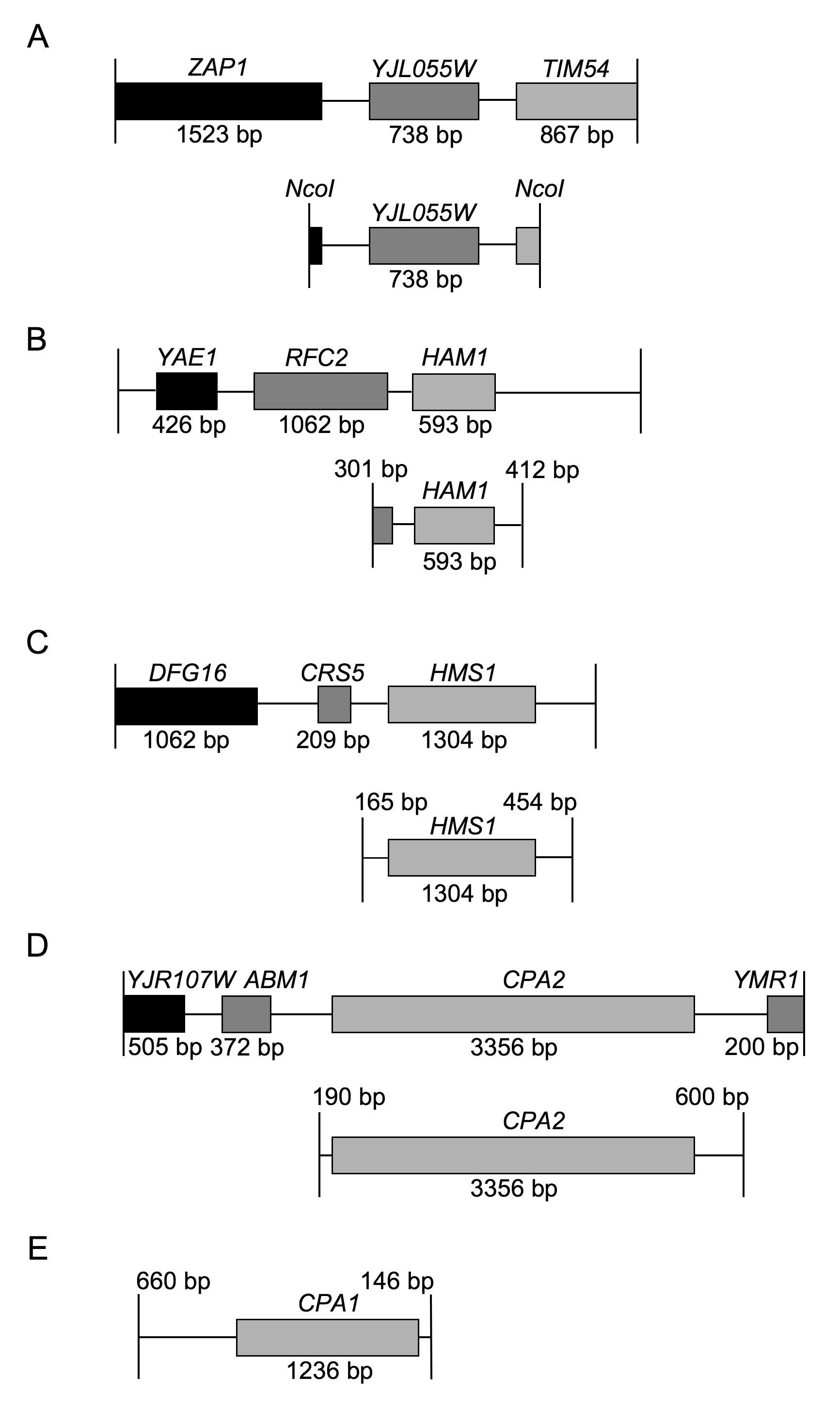

Supplement: Figure S1 — Restriction maps of inserts of the plasmids isolated in the 5-FU resistance screen. Open reading frames are shown as boxes. Below each insert, the shortest subclone that could still confer 5-FU resistance when overexpressed is shown. (TIF) [file pone.0052094.s001.tif]

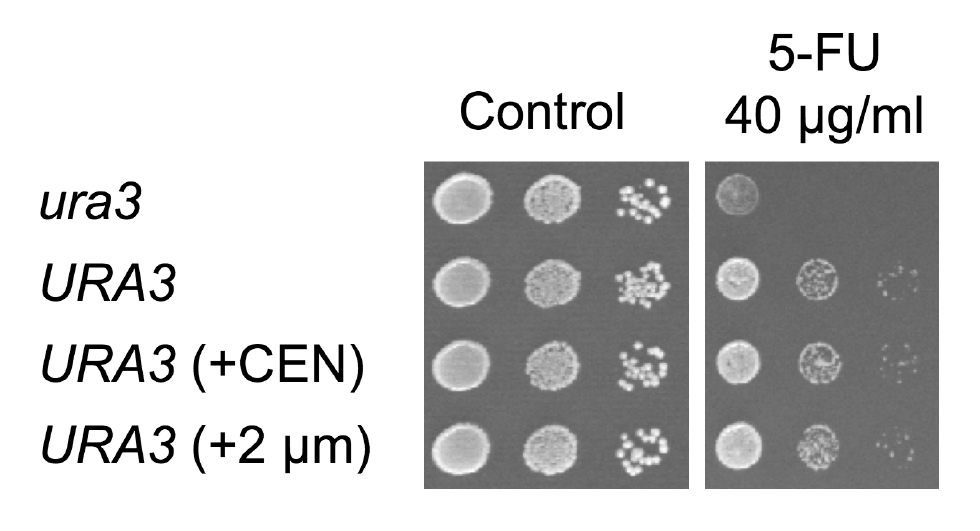

Supplement: Figure S2 — Effects of URA3 copy number on 5-FU sensitivity. Strains tested included the ura3 yeast strain BY4742, a URA3 transformant of BY4742 that carries the wild type gene at the URA3 locus, and BY4742 containing either the low copy number centromeric URA3 plasmid pFL39 or the high copy number 2 µm URA3 plasmid pFL44. The strains were grown in liquid medium to late exponential phase, serially diluted, and spotted onto synthetic complete media with or without 5-FU. Growth was scored after incubation at 30°C for four days. (TIF) [file pone.0052094.s002.tif]

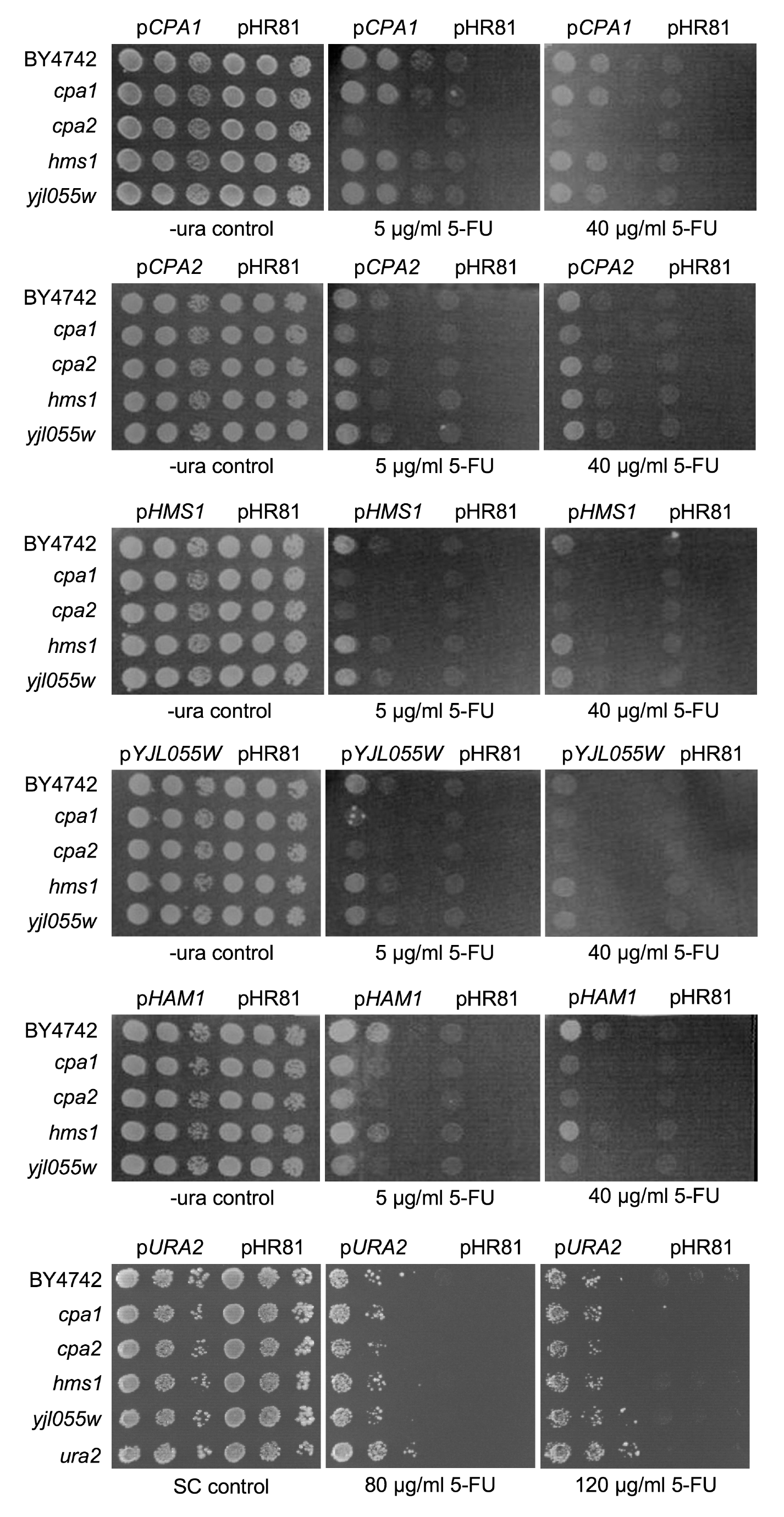

Supplement: Figure S3 — Cross-dependencies between different genes for the ability to confer 5-FU resistance. Each plasmid was transformed into yeast knockout strains where one of the other resistance genes had been deleted. Transformants were grown in liquid medium to late exponential phase, serially diluted, and spotted onto uracil-less plates with or without 5-FU at the indicated concentrations. In the bottom panes, synthetic complete media was used in order to permit growth of the control ura2 strain. Note that ammonium sulphate was used as nitrogen source, hence the dependency of CPA2 on CPA1, which provides ammonium ions to the CPA2 encoded enzyme, is only weakly detectable. (TIF) [file pone.0052094.s003.tif]

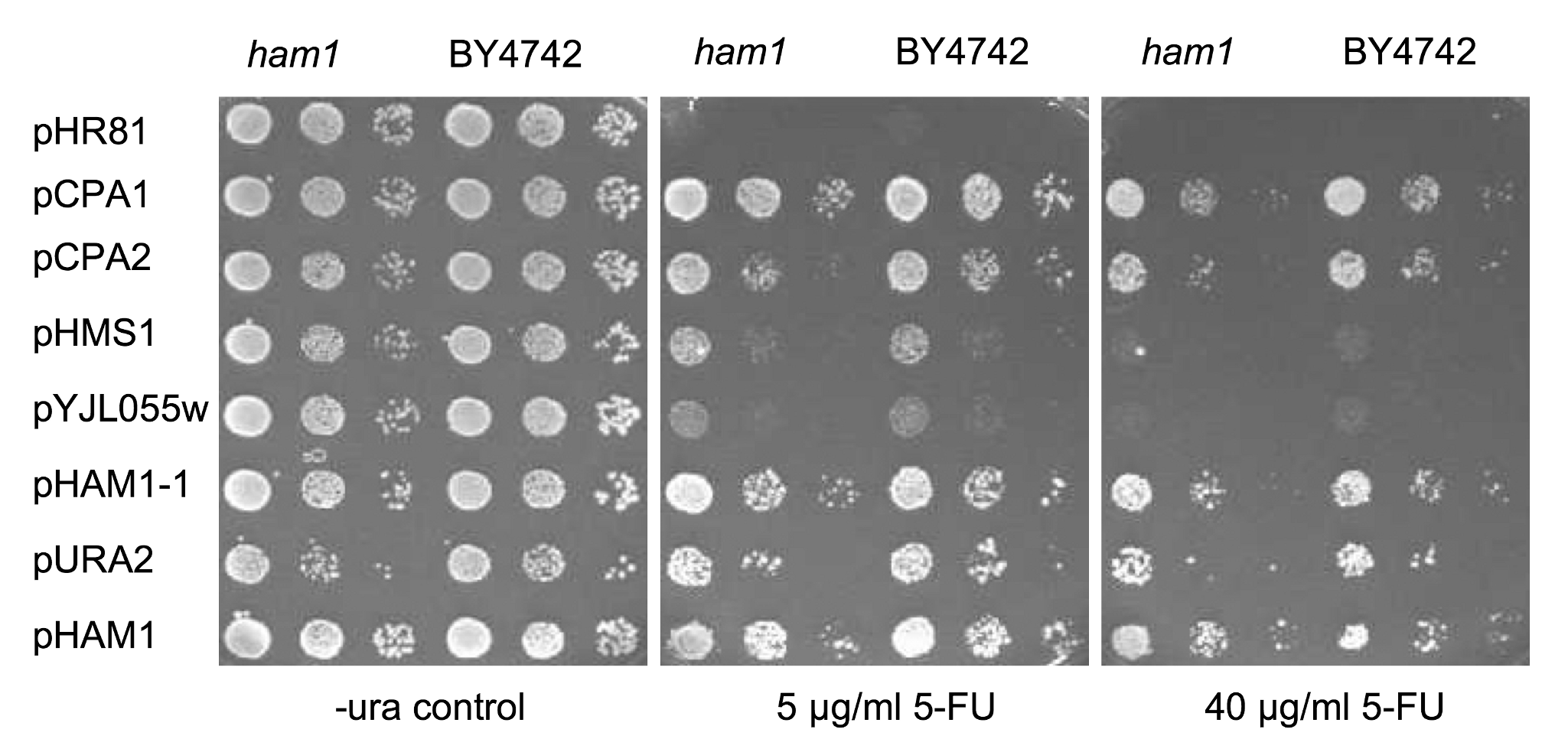

Supplement: Figure S4 — Test for dependencies of other genes on HAM1 for the ability to confer 5-FU resistance. Each plasmid was transformed into ham1 knockout and wild type strains. Transformants were grown in liquid medium to late exponential phase, serially diluted, and spotted onto uracil-less plates with or without 5-FU at the indicated concentrations. pHAM1-1 is a PCR subclone of pHAM1 containing sequences from 414 bp upstream of the HAM1 open reading frame to 301 bp downstream of the openreading frame. (TIF) [file pone.0052094.s004.tif]

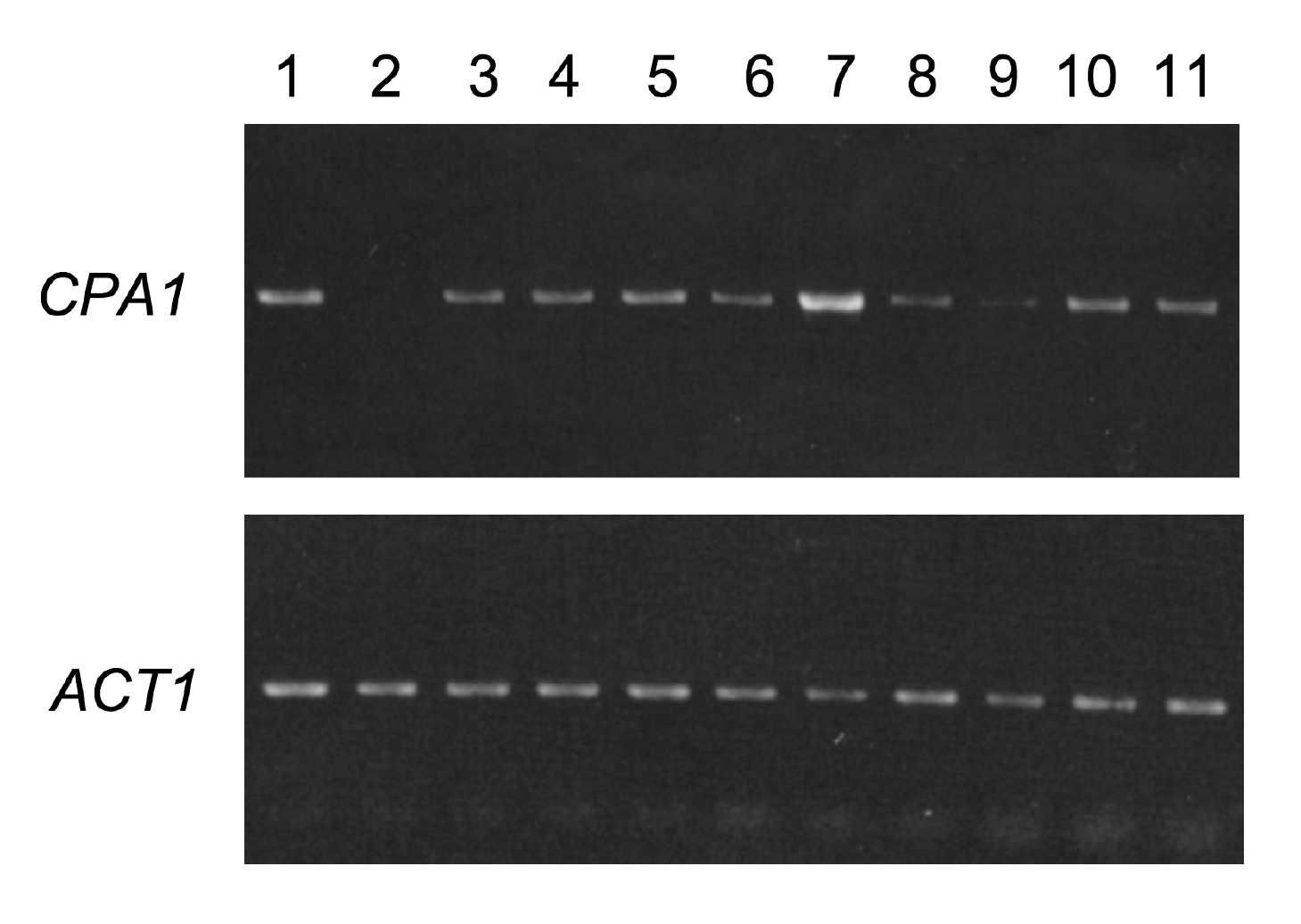

Supplement: Figure S5 — Analysis of CPA1 expression by reverse transcriptase-PCR. RNA was prepared from different knockout strains and the wild type BY4742 strain harbouring different plasmids. The RNA was used for reverse transcriptase-PCR of the CPA1 mRNA as described in Materials and Methods. The PCR products were separated on an agarose gel and visualized by ethidium bromide staining. As a control, we included the ACT1 mRNA encoding yeast actin. Lanes: 1, wild type; 2, cpa1 strain; 3, cpa2 strain; 4, hms1 strain; 5, yjl055w strain; 6, pHR81 (empty vector); 7, pCPA1; 8, pCPA2; 9, pHMS1; 10, pYJL055W; 11, pHAM1. (TIF) [file pone.0052094.s005.tif]
